# Combination strategies with PD-1/PD-L1 blockade: current advances and future directions

**DOI:** 10.1186/s12943-021-01489-2

**Published:** 2022-01-21

**Authors:** Ming Yi, Xiaoli Zheng, Mengke Niu, Shuangli Zhu, Hong Ge, Kongming Wu

**Affiliations:** 1 Department of Oncology, Tongji Hospital of Tongji Medical College, Huazhong University of Science and Technology, Wuhan, 430030 China; 2 Department of Radiation Oncology, The Affiliated Cancer Hospital of Zhengzhou University & Henan Cancer Hospital, Zhengzhou, 450008 China

**Keywords:** PD-1, PD-L1, Combination therapy, Angiogenesis inhibitor, Radiotherapy, STING, Gut microbiota, Bispecific antibody

## Abstract

Antibodies targeting programmed cell death protein-1 (PD-1) or its ligand PD-L1 rescue T cells from exhausted status and revive immune response against cancer cells. Based on the immense success in clinical trials, ten α-PD-1 (nivolumab, pembrolizumab, cemiplimab, sintilimab, camrelizumab, toripalimab, tislelizumab, zimberelimab, prolgolimab, and dostarlimab) and three α-PD-L1 antibodies (atezolizumab, durvalumab, and avelumab) have been approved for various types of cancers. Nevertheless, the low response rate of α-PD-1/PD-L1 therapy remains to be resolved. For most cancer patients, PD-1/PD-L1 pathway is not the sole speed-limiting factor of antitumor immunity, and it is insufficient to motivate effective antitumor immune response by blocking PD-1/PD-L1 axis. It has been validated that some combination therapies, including α-PD-1/PD-L1 plus chemotherapy, radiotherapy, angiogenesis inhibitors, targeted therapy, other immune checkpoint inhibitors, agonists of the co-stimulatory molecule, stimulator of interferon genes agonists, fecal microbiota transplantation, epigenetic modulators, or metabolic modulators, have superior antitumor efficacies and higher response rates. Moreover, bifunctional or bispecific antibodies containing α-PD-1/PD-L1 moiety also elicited more potent antitumor activity. These combination strategies simultaneously boost multiple processes in cancer-immunity cycle, remove immunosuppressive brakes, and orchestrate an immunosupportive tumor microenvironment. In this review, we summarized the synergistic antitumor efficacies and mechanisms of α-PD-1/PD-L1 in combination with other therapies. Moreover, we focused on the advances of α-PD-1/PD-L1-based immunomodulatory strategies in clinical studies. Given the heterogeneity across patients and cancer types, individualized combination selection could improve the effects of α-PD-1/PD-L1-based immunomodulatory strategies and relieve treatment resistance.

## Background

Programmed cell death 1 (PD-1) signaling is commonly hijacked by cancer cells to escape immune surveillance [1]. When PD-1 and T cell receptor (TCR) bind to their ligands, the immunoreceptor tyrosine-based inhibitory motif and immunoreceptor tyrosine-based switch motif of PD-1 are phosphorylated [2]. Subsequently, Src homology region 2 domain-containing phosphatase (SHP-2) is recruited and activated, reversing the phosphorylation of downstream signaling of TCR and CD28 [3, 4]. Besides inhibiting some early activating pathways of T cells, PD-1 directly undermines antigen recognition by disrupting the trimolecular interaction of TCR-pMHC-CD8 [5]. As a result, PD-1 signaling suppresses T cell functions, including activation, proliferation, and cytokine production [6]. At present, antibodies blocking PD-1 or its ligand PD-L1 have been approved to treat various solid and hematologic malignancies (Table 1) [7–12]. Although α-PD-1/PD-L1 therapies exhibit potent antitumor effects in some patients, most patients could not benefit from α-PD-1/PD-L1 treatments, owing to primary or acquired treatment resistance [13]. For the non-responders, PD-1 signaling is not the speed-limiting rheostat of cancer-immunity cycle, and it is insufficient to revive antitumor immunity by blocking PD-1 or PD-L1 [14].

**Table 1:** The approved indications of α-PD-1/PD-L1 antibodies in the globe

Drugs	Approval	SC	NSCLC	SCLC	RCC	HL	HNC	UC	CRC	HCC	ESC	MPM	GC	GEJC	TNBC	BC	CC	EC
Nivolumab	2014-US 2015-EU 2018-PRC	**√**	**√**	**√**	**√**	**√**	**√**	**√**	**√**	**√**	**√**	**√**	**√**	**√**	**–**	**–**	**–**	**–**
Pembrolizumab	2014-US 2015-EU 2018-PRC	**√**	**√**	**√**	**√**	**√**	**√**	**√**	**√**	**√**	**√**	**–**	**√**	**√**	**√**	**√**	**√**	**√**
Cemiplimab	2018-US 2019-EU	**√**	**√**	**–**	**–**	**–**	**–**	**–**	**–**	**–**	**–**	**–**	**–**	**–**	**–**	**–**	**–**	**–**
Toripalimab	2018-PRC	**√**	**–**	**–**	**–**	**–**	**√**	**√**	**–**	**–**	**–**	**–**	**–**	**–**	**–**	**–**	**–**	**–**
Sintilimab	2018-PRC	**–**	**√**	**–**	**–**	**√**	**–**	**–**	**–**	**√**	**–**	**–**	**–**	**–**	**–**	**–**	**–**	**–**
Camrelizumab	2019-PRC	**–**	**√**	**–**	**–**	**√**	**√**	**–**	**–**	**√**	**√**	**–**	**–**	**–**	**–**	**–**	**–**	**–**
Tislelizumab	2019-PRC	**–**	**√**	**–**	**–**	**√**	**–**	**√**	**–**	**–**	**–**	**–**	**–**	**–**	**–**	**–**	**–**	**–**
Zimberelimab	2021-PRC	**–**	**–**	**–**	**–**	**√**	**–**	**–**	**–**	**–**	**–**	**–**	**–**	**–**	**–**	**–**	**–**	**–**
Prolgolimab	2020-RU	**√**	**–**	**–**	**–**	**–**	**–**	**–**	**–**	**–**	**–**	**–**	**–**	**–**	**–**	**–**	**–**	**–**
Dostarlimab	2021-US 2021-EU	**–**	**–**	**–**	**–**	**–**	**–**	**–**	**–**	**–**	**–**	**–**	**–**	**–**	**–**	**–**	**–**	**√**
Atezolizumab	2016-US 2017-EU 2020-PRC	**√**	**√**	**√**	**–**	**–**	**–**	**√**	**–**	**√**	**–**	**–**	**–**	**–**	**√**	**–**	**–**	**–**
Durvalumab	2017-US 2018-EU 2019-PRC	**–**	**√**	**√**	**–**	**–**	**–**	**–**	**–**	**–**	**–**	**–**	**–**	**–**	**–**	**√**	**–**	**–**
Avelumab	2017-US 2017-EU	**√**	**–**	**–**	**√**	**–**	**–**	**√**	**–**	**–**	**–**	**–**	**–**	**–**	**–**	**–**	**–**	**–**

Abbreviations: *SC* skin cancer, *NSCLC* non-small cell lung cancer, *RCC* renal cell carcinoma, *HL* Hodgkin lymphoma, *HNC* head and neck cancer, *UC* urothelial carcinoma, *CRC* colorectal cancer, *HCC* hepatocellular carcinoma, *ESC* esophageal carcinoma, *MPM* malignant pleural mesothelioma, *GC* gastric cancer, *GEJC* gastroesophageal junction cancer, *TNBC* triple-negative breast cancer, *BC* bladder cancer, *CC* cervical cancer, *EC* endometrial cancer, *EU* European Union, *PRC* People’s Republic of China. √ denotes the indication approved in the globe

Apart from PD-1 signaling, other immune checkpoints, abnormal angiogenesis, immunosuppressive immune cells or cytokines, cancer-associated adipocytes, and hyperactive cancer-associated fibroblasts also modulate cancer-immune set point and promote immune tolerance [15–20]. Logically, removing these negative factors could enhance the therapeutic effect of α-PD-1/PD-L1 and relieve drug resistance. On the other hand, some positive factors such as immunogenic cancer cell death, immunosupportive cytokines, and professional antigen presentation cells (pAPCs) contribute to immune clearance [21]. Correspondingly, strengthening these positive elements might boost the cancer-immune cycle, drive the transformation from cold to hot tumors, and improve the response to α-PD-1/PD-L1 therapies [21].

The combination strategy is deemed as a rational and feasible approach to achieve optimal treatment effects. Accumulating evidence indicates that chemotherapy, radiotherapy, angiogenesis inhibitor, stimulator of interferon genes (STING) agonist, fecal microbiota transplantation (FMT), epigenetic modulators, or other immunomodulators could synergize α-PD-1/PD-L1, by enhancing cancer antigen release, APC function, or effector activity [22–31]. In this review, we summarized the synergistic effects of combination immunotherapies and the underlying mechanisms. Moreover, given the development of antibody technology, we also introduced the emerging bispecific or bifunctional antibodies targeting PD-1 or PD-L1.

## Conventional chemotherapy combined with α-PD-1/PD-L1

### Chemotherapy modifying the TME

**Fig. 1:**
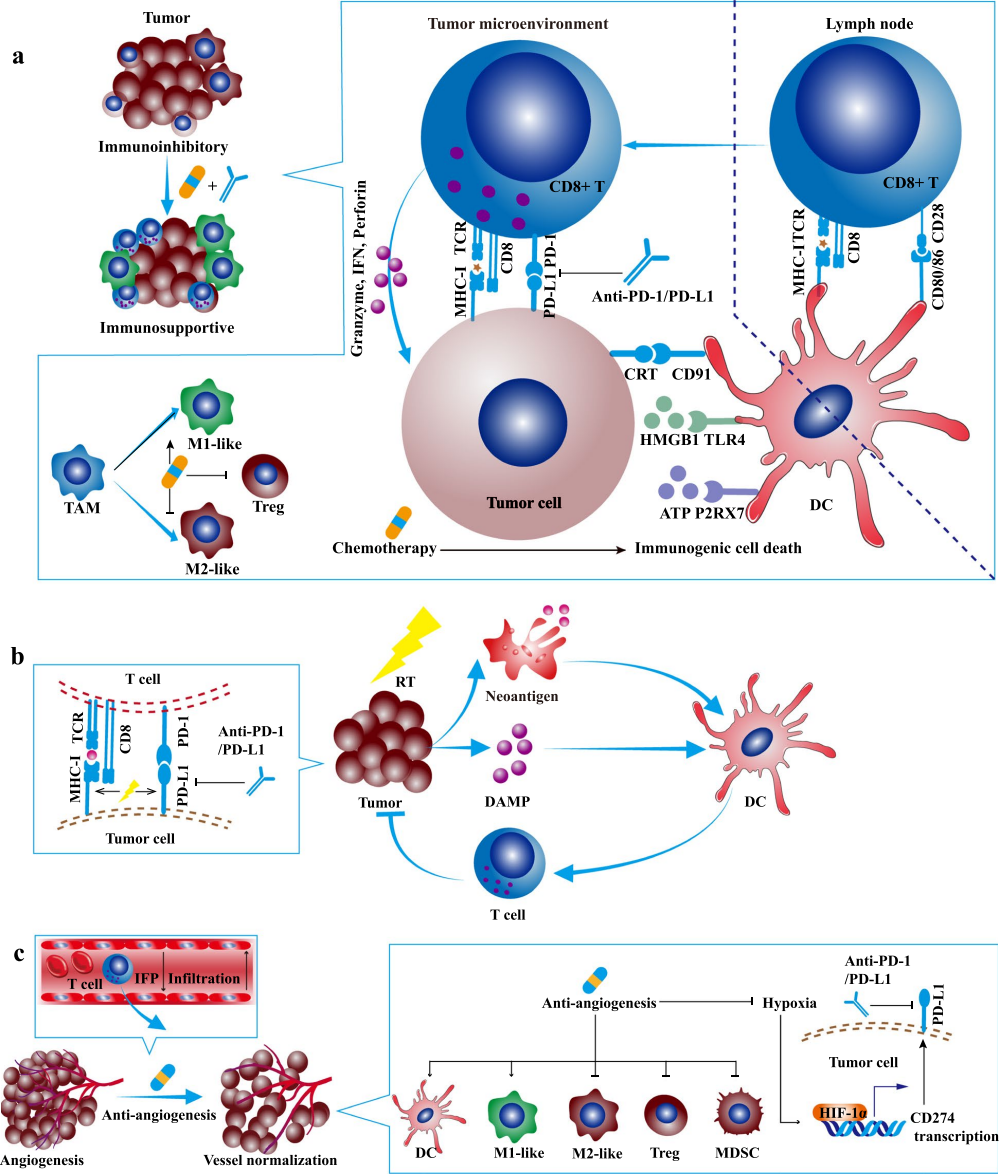
The synergistic antitumor efficacies and mechanisms of α-PD-1/PD-L1 in combination with chemotherapy, radiotherapy, or angiogenesis inhibitor. **a** Chemotherapy synergizes with α-PD-1/PD-L1. Some cytotoxic chemotherapeutic drugs could induce immunogenic cell death and stimulate antitumor immune response. Immunogenic cell death is featured with some upregulated damage-associated molecular patterns (DAMPs) such as calreticulin (CRT), ATP, and high-mobility group box 1 (HMGB1). The ATP-P2RX7, CRT-CD91, and HMGB1-TLR4 pathways facilitate the antigen capture and presentation of DC, ultimately motivating adaptive antitumor immune response. Apart from immunogenic cell death, low-dose chemotherapy depletes regulatory T cells (Tregs) and promotes the repolarization of tumor-associated macrophage (TAM) from M2-like to M1-like phenotype. **b** Radiotherapy synergizes with α-PD-1/PD-L1. Firstly, radiotherapy could induce immunogenic cell death, enhance antitumor immune response, promote T cell infiltration, expand T-cell receptor (TCR) repertoire in the TME. Secondly, radiotherapy upregulates the expression of PD-L1 on tumor cells, which might be utilized by additional α-PD-1/PD-L1. Thirdly, radiotherapy increases the MHC-I on tumor cells and relieves resistance to α-PD-1/PD-L1. **c** Angiogenesis inhibitor synergizes with α-PD-1/PD-L1. Angiogenesis inhibitor blocks proangiogenic pathways, promotes vessel normalization, improves tumor perfusion and oxygenation, restores the hypoxic TME, and enhances drug delivery. Also, angiogenesis inhibitor reshapes the TME: promoting T cell infiltration and DC maturation, enhancing the differentiation towards M1-like macrophage, decreasing the ratio of Treg and MDSC, and alleviating hypoxia-induced PD-L1

Chemotherapy retards tumor growth mainly by arresting cell cycle, inhibiting DNA replication, disturbing cell metabolism, or suppressing microtubule assembly [32]. Besides, some cytotoxic chemotherapeutic drugs such as anthracycline and oxaliplatin could induce immunogenic cell death and stimulate antitumor immune response [33, 34]. Immunogenic cell death is featured with some upregulated damage-associated molecular patterns (DAMPs) such as the secretion of IFN-I, the exposure of endoplasmic reticulum proteins especially calreticulin (CRT, an eat-me signal) on cell membrane, the leak of ATP (a find-me signal), and the release of high-mobility group box 1 (HMGB1) [35]. The receptors of CRT, ATP, and HMGB1 are CD91, P2RX7, TLR4 on dendritic cells (DCs). The ATP**-**P2RX7 signaling recruits DCs into the tumor bed; the CRT-CD91 axis promotes DC to engulf cancer antigens; the HMGB1-TLR4 pathway facilitates the optimal cancer antigen presentation [36]. Collectively, the antigen capture and presentation of DC are enhanced, ultimately motivating adaptive antitumor immune response (Fig. 1a).

Apart from immunogenic cell death, chemotherapy could directly eliminate immune suppressor cells and enhance the functions of effector cells, especially administrated at the dose below maximum-tolerated dose [37]. Low-dose cyclophosphamide depleted circulating and tumor-infiltrating regulatory T cells (Tregs) [38–40]. Moreover, paclitaxel promoted tumor-associated macrophage (TAM) to repolarize from M2-like to M1-like phenotype [41]. Notably, although 5-fluorouracil, doxorubicin, gemcitabine, and docetaxel reduced circulating myeloid-derived suppressor cells (MDSCs) in mouse models [42–45], some chemotherapeutic agents increased circulating MDSCs in cancer patients [46]. Therefore, the chemotherapy-mediated MDSC depletion remains further verification in cancer patients. Besides suppressor cells, some certain chemotherapies such as cyclophosphamide, gemcitabine, and vinblastine recruited and activated DC in the immunogenic cell death manner [47–49]. Also, chemotherapeutic drugs such as vinblastine, 5-fluorouracil, and oxaliplatin, could directly enhance the functions of DC and promote IL-12 secretion [49, 50]. Additionally, pemetrexed enhanced the activation of tumor-infiltrating lymphocytes (TILs) by improving mitochondrial biogenesis, independent of immunogenic cell death [51].

### Chemotherapy combined with α-PD-1

Based on the immune-modulatory effect of chemotherapeutic agents, chemotherapy might be an appropriate partner with α-PD-1/PD-L1 to achieve both rapid and long-term cancer control. Nowadays, chemotherapy combined with α-PD-1/PD-L1 has become a standard-of-care option for some cancer patients, and there are hundreds of ongoing clinical trials exploring the efficacy and safety of chemotherapy plus α-PD-1/PD-L1 (Table 2). In the clinical trial KEYNOTE-021 (phase 2), non-squamous non-small cell lung cancer (NSCLC) patients receiving pembrolizumab combined with standard chemotherapy (carboplatin and pemetrexed) had a higher response rate and longer progression-free survival (PFS) than did patients receiving standard chemotherapy [52]. Based on the results of KEYNOTE-021, pembrolizumab plus chemotherapy has been approved by the FDA as the first-line treatment for advanced non-squamous NSCLC, regardless of PD-L1 level [52]. Later, in two phase 3 clinical studies (KEYNOTE-189 and KEYNOTE-407), pembrolizumab combined with standard chemotherapy led to a better overall survival (OS) and PFS in NSCLC patients, relative to chemotherapy monotherapy [53, 54]. The results of KEYNOTE-407 engaged the FDA to approve pembrolizumab combined with chemotherapy for squamous NSCLC in 2018. Then, based on a string of successes (KEYNOTE-355, KEYNOTE-590, and KEYNOTE-811), the indication of pembrolizumab plus chemotherapy was expanded to advanced triple-negative breast cancer (TNBC), esophageal cancer, gastroesophageal junction cancer (GEJC) [55–57].

**Table 2:** The clinical trials exploring the efficacy of α-PD-1/PD-L1 plus chemotherapy

Clinical trial	Phase	α-PD-1/PD-L1	Chemotherapy	Cancer type	Primary outcome measures
NCT02039674	1/2	Pembrolizumab	Pemetrexed and carboplatin	Non-squamous NSCLC	ORR
NCT02775435	3	Pembrolizumab	Paclitaxel/nab-paclitaxel and carboplatin	Squamous NSCLC	PFS, OS
NCT02819518	3	Pembrolizumab	Paclitaxel; nab-paclitaxel; gemcitabine and carboplatin	TNBC	PFS, OS
NCT03189719	3	Pembrolizumab	Cisplatin and 5-fluorouracil	Esophageal or GEJ cancer	PFS, OS
NCT03615326	3	Pembrolizumab	Trastuzumab plus either 5-fluorouracil plus cisplatin or capecitabine plus oxaliplatin	HER2+ gastric or GEJ adenocarcinoma	PFS, OS
NCT02872116	3	Nivolumab	5-fluorouracil and leucovorin plus oxaliplatin; capecitabine and oxaliplatin	Gastric cancer, esophageal or GEJ adenocarcinoma	PFS, OS
NCT03607539	3	Sintilimab	Pemetrexed and platinum	Non-squamous NSCLC	PFS
NCT03629925	3	Sintilimab	Gemcitabine and platinum	Squamous NSCLC	PFS
NCT03134872	3	Camrelizumab	Carboplatin and pemetrexed	Non-squamous NSCLC	PFS
NCT03707509	3	Camrelizumab	Gemcitabine and cisplatin	Nasopharyngeal carcinoma	PFS
NCT03594747	3	Tislelizumab	Paclitaxel/nab-paclitaxel and carboplatin	Squamous NSCLC	PFS
NCT03663205	3	Tislelizumab	Platinum and pemetrexed	Non-squamous NSCLC	PFS
NCT02366143	3	Atezolizumab	Bevacizumab plus paclitaxel and carboplatin	Non-squamous NSCLC	PFS, OS
NCT02763579	3	Atezolizumab	Carboplatin and etoposide	SCLC	PFS, OS
NCT02425891	3	Atezolizumab	Nab-paclitaxel	TNBC	PFS, OS
NCT02367781	3	Atezolizumab	Carboplatin and nab-paclitaxel	Non-squamous NSCLC	PFS, OS
NCT03043872	3	Durvalumab	Etoposide and carboplatin/ cisplatin	SCLC	OS

Abbreviations: *NSCLC* non-small cell lung cancer, *ORR* objective response rate, *PFS* progression-free survival, *OS* overall survival, *GEJ* gastroesophageal junction, *SCLC* small cell lung cancer

Generally, pembrolizumab has a great advantage on chemoimmunotherapy, with a broad range of indications. The FDA rarely approves chemoimmunotherapeutic strategies with other α-PD-1 drugs (except for nivolumab combined with chemotherapy for gastric cancer and GEJC) [58]. In China, the NMPA approved sintilimab plus pemetrexed and platinum as the first-line treatment for advanced non-squamous NSCLC, based on the results of ORIENT-11 [59]. In addition, the NMPA approved sintilimab plus gemcitabine and platinum as the first-line treatment for advanced squamous NSCLC, based on the results of ORIENT-12 [60]. In 2020, the NMPA also approved camrelizumab plus carboplatin and pemetrexed as the first-line treatment for non-squamous NSCLC, based on the results of CameL [61]. Later in 2021, the NMPA approved camrelizumab plus gemcitabine and cisplatin (for advanced nasopharyngeal carcinoma) and tislelizumab plus chemotherapy (for NSCLC) [62–64].

### Chemotherapy combined with α-PD-L1

Besides α-PD-1, α-PD-L1-based chemoimmunotherapy also attracts intensive attention, especially chemoimmunotherapeutic regimens with atezolizumab. IMpower150 is the pioneer of this series of studies, assessing the efficacy of atezolizumab plus angiogenesis inhibitor and chemotherapy in advanced non-squamous NSCLC [65]. Based on the results of IMpower150, the FDA approved atezolizumab plus bevacizumab, paclitaxel, and carboplatin as the first-line treatment for advanced non-squamous NSCLC [65]. Subsequently, the FDA approved atezolizumab plus chemotherapy for TNBC (atezolizumab plus nab-paclitaxel, based on IMpassion130), SCLC (atezolizumab plus carboplatin and etoposide, based on IMpower133), and non-squamous NSCLC (atezolizumab plus nab-paclitaxel and carboplatin, based on IMpower130) [66–68]. Moreover, based on the results of CASPIAN, durvalumab combined with platinum plus etoposide therapy was approved for SCLC in the US [69]. Presently, there are still dozens of chemoimmunotherapeutic regimens with α-PD-1/PD-L1 awaiting approval in the US and China.

## Radiotherapy combined with α-PD-1/PD-L1

### The mechanisms by which radiotherapy synergizing α-PD-1/PD-L1

Like some chemotherapeutic drugs, radiotherapy could induce immunogenic cell death and enhance antitumor immune response [70]. On the one hand, immunogenic cell death-associated DAMPs and cytokines especially IFN-I recruit immune cells and promote the function of DCs. On the other hand, released tumor antigens could be captured by DCs and presented to T cells [70]. Consequently, radiotherapy not only eliminates local lesions but also stimulates the systemic antitumor immune response (also known as abscopal effects) [71]. Previous preclinical and clinical studies demonstrated that radiotherapy could synergize α-PD-1/PD-L1 in multiple manners. Firstly, radiotherapy promoted T cell infiltration, increased the number of TILs, and expanded T-cell receptor (TCR) repertoire in the TME [72, 73]. Secondly, radiotherapy upregulated the expression of PD-L1 on tumor cells, which can be utilized by additional α-PD-1/PD-L1 [74]. Thirdly, radiotherapy increased the MHC-I on tumor cells and relieved resistance to α-PD-1/PD-L1 (Fig. 1b) [75]. However, some problems have not been well addressed, including the fractionation, dose, schedule of radiotherapy, irradiated tumor volume, irradiated regional lymph nodes, and the schedule of α-PD-1/PD-L1 post-radiotherapy [76].

### Clinical studies exploring the efficacy and safety of radiotherapy combined with α-PD-1/PD-L1

Most radioimmunotherapy regimens are based on stereotactic body radiotherapy (SBRT), which could precisely deliver ablative doses of radiation in image-guided and intensity-modulated manners [77]. The results of the phase 1 study NCT02608385 demonstrated that α-PD-1/PD-L1 combined with SBRT was well-tolerable (Table 3) [24]. Moreover, the results of some phase 1/2 studies (NCT02621398, NCT02434081, NCT02586207, NCT02383212, and NCT02402920) showed that α-PD-1/PD-L1 plus chemoradiotherapy was tolerable in advanced NSCLC, head and neck squamous cell carcinoma (HNSCC), and SCLC patients, with promising clinical outcomes [78–82].

**Table 3:** The clinical trials exploring the efficacy of α-PD-1/PD-L1 plus radiotherapy

Clinical trial	Phase	α-PD-1/PD-L1	Radiotherapy	Cancer type	Primary outcome measures
NCT02608385	1	Pembrolizumab	SBRT	Solid tumors	Recommended SBRT dose
NCT02621398	1	Pembrolizumab	Concurrent chemoradiation	NSCLC	MTD and DLT
NCT02434081	2	Nivolumab	Concurrent chemoradiation	NSCLC	Safety
NCT02586207	1	Pembrolizumab	Concurrent chemoradiation	HNSCC	Safety
NCT02383212	1	Cemiplimab	Concurrent chemoradiation	Solid tumors	Safety, DLT
NCT02402920	1	Pembrolizumab	Concurrent chemoradiation; Concurrent radiation	SCLC	DLT
NCT02904954	2	Durvalumab	SBRT	NSCLC	Pathological response rate
NCT02125461	3	Durvalumab	Concurrent chemoradiation	NSCLC	PFS, OS
NCT02684253	2	Nivolumab	SBRT	HNSCC	BOR
NCT02952586	3	Avelumab	Concurrent chemoradiation	HNSCC	PFS

Abbreviations: *SBRT* stereotactic body radiotherapy, *NSCLC* non-small cell lung cancer, *MDT* maximum tolerated dose, *DLT* dose limiting toxicity, *PFS* progression-free survival, *OS* overall survival, *SCLC* small cell lung cancer, *HNSCC* head and neck squamous cell carcinoma, *BOR* best overall response

In the phase 2 study NCT02904954, SBRT combined with durvalumab acquired a superior antitumor effect to durvalumab in early-stage NSCLC [83]. In the combination therapy arm, patients received 24 Gy SBRT before durvalumab treatment (given in three consecutive daily fractions of 8 Gy) [83]. The major pathological response rate was significantly higher in the SBRT combined with durvalumab arm than that in the durvalumab arm [83]. Additionally, the results of the phase 3 study NCT02125461 indicated that sequential durvalumab treatment markedly improved the PFS and OS of NSCLC patients undergoing chemoradiotherapy [84]. However, in the phase 2 study NCT02684253, SBRT combined with nivolumab was not superior to nivolumab in response rate, PFS, and OS in advanced HNSCC [85]. Furthermore, in the phase 3 study NCT02952586 exploring the efficacy of avelumab plus standard-of-care chemoradiotherapy in HNSCC, it did not meet the primary endpoint (PFS) [86]. Considering the multiple variants in the combination therapy such as dose, volume, fractionation, sequence, more efforts are needed to explore optimal radioimmunotherapy schemes.

## Angiogenesis inhibitor combined with α-PD-1/PD-L1

### Abnormal angiogenesis hampering the antitumor immune response

Hyperactive metabolism and incommensurate blood supply contribute to the hypoxic and acid TME [87]. As the feedback on hypoxia, the levels of some pro-angiogenic cytokines such as vascular endothelial growth factor (VEGF) and angiopoietin 2 (ANGPT2) are upregulated, driving angiogenesis [88]. The disorganized angiogenesis promotes the formation of the immunosuppressive TME [16]. Firstly, the immature and leaky vessels lead to increased interstitial fluid pressure, which hinders blood perfusion and immune cell infiltration [89]. Secondly, VEGF could inhibit the maturation of DC, induce the exhaustion of T cells, promote the proliferation of Tregs, and increase the ratio of MDSCs [90–93]. Thirdly, despite without direct influence on T cells, ANGPT2 recruits Tie-2-expressing monocytes, enhances the differentiation towards M2-like macrophages, and upregulates the expression of IL-10 [94–97]. Moreover, other proangiogenic cytokines such as placental growth factor (PLGF) and TGF-β also contribute to immunosuppression [98].

### Angiogenesis inhibitor synergizing with α-PD-1/PD-L1

Commonly, the transformation from nascent to functional vessel needs maturational processes, which are disturbed by hyperactive angiogenesis in the TME [99]. Angiogenesis inhibitor blocks these proangiogenic pathways, promotes vessel normalization, improves tumor perfusion and oxygenation, restores the hypoxic TME, and enhances drug delivery [100, 101]. Also, angiogenesis inhibitor reshapes the TME: promoting T cell infiltration and DC maturation, enhancing the differentiation towards M1-like macrophage, decreasing the ratio of Treg and MDSC, and alleviating hypoxia-induced PD-L1 (Fig. 1c) [93, 102–105]. In the multiple preclinical studies, angiogenesis inhibitor enhanced the efficacy of α-PD-1/PD-L1 in murine tumor models [106–108].

In 2019, pembrolizumab combined with axitinib was approved by the FDA as the first-line treatment for advanced RCC, based on the results of KEYNOTE-426 (Table 4) [109]. At a median follow-up time of 30.6 months, the median OS and PFS were longer in the pembrolizumab combined with axitinib arm compared to those in the sunitinib arm [109]. Moreover, pembrolizumab plus lenvatinib was also approved for advanced endometrial carcinoma [110]. Additionally, as mentioned above, the FDA approved atezolizumab plus bevacizumab and chemotherapy as the first-line treatment for advanced non-squamous NSCLC based on the results of IMpower150 [65]. Then, in 2020, the FDA approved atezolizumab combined with bevacizumab for advanced HCC based on the data of IMbrave150 [111]. Besides pembrolizumab plus axitinib, nivolumab plus cabozantinib (based on CheckMate-9ER) [112] and avelumab plus axitinib (based on JAVELIN Renal 101) [113] were also approved by the FDA as the initial-line treatment for RCC.

**Table 4:** The clinical trials exploring the efficacy of α-PD-1/PD-L1 combined with angiogenesis inhibitor

Clinical trial	Phase	α-PD-1/PD-L1	Angiogenesis inhibitor	Cancer type	Primary outcome measures
NCT02853331	3	Pembrolizumab	Axitinib	RCC	PFS, OS
NCT02501096	1b/2	Pembrolizumab	Lenvatinib	Solid tumors	MTD, ORR, DLT
NCT03517449	3	Pembrolizumab	Lenvatinib	Endometrial cancer	PFS, OS
NCT02366143	3	Atezolizumab	Bevacizumab plus chemotherapy	Non-Squamous NSCLC	PFS, OS
NCT03434379	3	Atezolizumab	Bevacizumab	HCC	PFS, OS
NCT03141177	3	Nivolumab	Cabozantinib	RCC	PFS
NCT02684006	3	Avelumab	Axitinib	RCC	PFS, OS
NCT03628521	1b	Sintilimab	Anlotinib	NSCLC	Safety, ORR
NCT03794440	2/3	Sintilimab	IBI305	HCC	PFS, OS
NCT02942329	1/2	Camrelizumab	Apatinib	Gastric cancer, HCC	OS rate
NCT03417895	2	Camrelizumab	Apatinib	SCLC	Safety, ORR
NCT03816553	2	Camrelizumab	Apatinib	Cervical cancer	ORR
NCT03394287	2	Camrelizumab	Apatinib	TNBC	ORR
NCT03359018	2	Camrelizumab	Apatinib	Osteosarcoma	PFS, CBR
NCT03086174	1	Toripalimab	Axitinib	Kidney cancer, melanoma	Safety

Abbreviations: *NSCLC* non-small cell lung cancer, *MDT* maximum tolerated dose, *DLT* dose limiting toxicity, *PFS* progression-free survival, *OS* overall survival, *SCLC* small cell lung cancer, *NSCLC* non-small cell lung cancer, *RCC* renal cell carcinoma, *TNBC* triple-negative breast cancer, *HCC* hepatocellular carcinoma, *CBR* clinical benefit rate

Up to now, most angiogenesis inhibitor plus α-PD-1/PD-L1 strategies are undergoing clinical trials, having not been approved by the FDA or NMPA. Combination therapies such as sintilimab plus anlotinib, sintilimab plus IBI305 (bevacizumab biosimilar), camrelizumab plus apatinib, and toripalimab plus axitinib demonstrated potent antitumor effects in multiple types of cancers [22, 114–120]. Despite encouraging results, further phase 3 trials are needed to validate the efficacies of these combination regimens.

## Dual immune checkpoint blockade or co-stimulatory molecule agonist plus α-PD-1/PD-L1

### α-CTLA-4 plus α-PD-1/PD-L1

CTLA-4 is primarily expressed on activated T cells and Tregs, as a negative regulator for T cell activation [121]. On the one hand, CTLA-4 could competitively suppress the binding of CD28 to CD80/CD86, halting the secondary signal of T cell activation [122]. On the other hand, CTLA-4 engagement with CD80/CD86 counteracts TCR-induced downstream signaling and suppresses PI3K-Akt pathway (vital signaling of T cell activation), via SHP-2 and protein phosphatase 2A (PP2A) [123, 124]. Additionally, CTLA-4 could capture, remove, and degrade its ligands CD80/CD86 from nearby APCs by trans-endocytosis, further hampering the co-stimulatory signal [125]. It is commonly believed that CTLA-4 signaling mainly undermines T cell priming in secondary lymphoid organs [126]. Ipilimumab (developed by Bristol-Myers Squibb) is the first approved α-CTLA-4 drug, initially used for advanced melanoma [127]. So far, the mechanism of antitumor activity of ipilimumab is still unclear. Theoretically, ipilimumab blocks the binding of CTLA-4 to CD80/CD86, removes the immunoinhibitory signal, and promotes T cell priming. However, multiple studies have been confirmed that antibody-dependent cell-mediated cytotoxicity of Treg also substantially contributes to the antitumor activity of ipilimumab [128–130].

In the clinic, ipilimumab is rarely used alone. Instead, ipilimumab is commonly used in combination with nivolumab. Although both CTLA-4 and PD-1 are immune checkpoints, they inhibit T cell activation in nonredundant manners. Therefore, α-CTLA-4 might cooperate with α-PD-1/PD-L1 to boost the antitumor immune response. Accumulating evidence has indicated that dual PD-1/PD-L1 and CTLA-4 blockade has superior antitumor activity in some types of cancers [131]. The results of CheckMate-069, CheckMate-067, and CheckMate-142 showed that ipilimumab plus nivolumab significantly improved the outcomes of patients, relative to ipilimumab or nivolumab monotherapies [132–134]. Moreover, the data of CheckMate-214, CheckMate-227, and CheckMate-743 indicated the superior efficacy of ipilimumab plus nivolumab over the standard targeted therapy or chemotherapy [135–137]. Until now, the FDA has approved the ipilimumab plus nivolumab for melanoma, RCC, MSI-H/dMMR colorectal cancer, HCC, PD-L1 positive NSCLC, and malignant pleural mesothelioma (Table 5) [132–138].

**Table 5:** The clinical trials exploring the efficacy of dual immune checkpoint blockade or immune checkpoint agonist plus α-PD-1/PD-L1

Clinical trial	Phase	α-PD-1/PD-L1	Other immune checkpoint inhibitors (Target)	Cancer type	Primary outcome measures
NCT01844505	3	Nivolumab	Ipilimumab (CTLA-4)	Melanoma	PFS, OS, PFS rate, OS rate
NCT01927419	2	Nivolumab	Ipilimumab (CTLA-4)	Melanoma	ORR
NCT02060188	2	Nivolumab	Ipilimumab (CTLA-4)	Colorectal cancer	ORR
NCT02231749	3	Nivolumab	Ipilimumab (CTLA-4)	RCC	ORR, PFS, OS
NCT02477826	3	Nivolumab	Ipilimumab (CTLA-4)	NSCLC	PFS, OS
NCT02899299	3	Nivolumab	Ipilimumab (CTLA-4)	Mesothelioma	OS
NCT03043872	3	Durvalumab	Tremelimumab (CTLA-4) plus chemotherapy	SCLC	OS
NCT02812420	1	Durvalumab	Tremelimumab (CTLA-4)	Urothelial cancer	Safety
NCT02516241	3	Durvalumab	Tremelimumab (CTLA-4)	Urothelial cancer	OS
NCT02870920	2	Durvalumab	Tremelimumab (CTLA-4)	Colorectal cancer	OS
NCT02369874	3	Durvalumab	Tremelimumab (CTLA-4)	HNSCC	OS
NCT02453282	3	Durvalumab	Tremelimumab (CTLA-4)	NSCLC	OS, PFS
NCT02352948	2	Durvalumab	Tremelimumab (CTLA-4)	NSCLC	OS, PFS
NCT02340975	1/2	Durvalumab	Tremelimumab (CTLA-4)	Gastric or GEJ adenocarcinoma	Safety, ORR, PFS rate
NCT02319044	2	Durvalumab	Tremelimumab (CTLA-4)	HNSCC	ORR
NCT03081923	2	Durvalumab	Tremelimumab (CTLA-4)	Germ cell tumors	ORR
NCT02588131	2	Durvalumab	Tremelimumab (CTLA-4)	Mesothelioma	ORR
NCT02519348	1/2	Durvalumab	Tremelimumab (CTLA-4)	HCC	Safety
NCT02558894	2	Durvalumab	Tremelimumab (CTLA-4)	Pancreatic ductal adenocarcinoma	ORR
NCT03099109	1	LY3300054	LY3321367 (TIM-3)	Solid tumor	Safety
NCT02791334	1	LY3300054	LY3321367 (TIM-3)	Solid tumor	Safety
NCT02608268	1/2	Spartalizumab	Sabatolimab (TIM-3)	Solid tumor	Safety, ORR
NCT03470922	2/3	Nivolumab	Relatlimab (LAG-3)	Melanoma	PFS
NCT03667716	1	Nivolumab	COM701 (PVRIG)	Solid tumor	Safety
NCT03563716	2	Atezolizumab	Tiragolumab (TIGIT)	NSCLC	ORR, PFS
NCT02179918	1	Pembrolizumab	PF-05082566 (4-1BB)	Solid tumor	Safety
NCT03502330	1	Nivolumab	APX005M (CD40)	Melanoma, NSCLC, RCC	Safety
NCT03829501	1/2	Atezolizumab	KY1044 (ICOS)	Solid tumor	Safety, ORR
NCT02740270	1	Spartalizumab	GWN323 (GITR)	Solid tumor, lymphomas	Safety

Abbreviations: *NSCLC* non-small cell lung cancer, *PFS* progression-free survival, *OS* overall survival, *SCLC* small cell lung cancer, *NSCLC* non-small cell lung cancer, *RCC* renal cell carcinoma, *HCC* hepatocellular carcinoma, *GEJ* gastroesophageal junction, *HNSCC* head and neck squamous cell carcinoma

Tremelimumab is a human IgG2 monoclonal antibody (developed by AstraZeneca) targeting CTLA-4, which has entered phase 3 clinical trials [139]. The efficacy of tremelimumab plus durvalumab has been intensively investigated in SCLC, urothelial carcinoma, colorectal cancer, HNSCC, NSCLC, gastric and GEJ adenocarcinoma, germ cell tumors, mesothelioma, pancreatic ductal adenocarcinoma, and HCC [69, 140–152]. The results of some clinical trials were unsatisfactory, and no additional benefit was brought by tremelimumab plus durvalumab, compared to durvalumab monotherapy or standard chemotherapy [140, 142, 144]. However, the subgroup analysis showed that tremelimumab plus durvalumab markedly improved the OS of NSCLC patients with a high tumor mutation burden [145], indicating the importance of appropriate patient selection for the optimal benefit of tremelimumab plus durvalumab.

Apart from efficacy, it is concerned that dual PD-1/PD-L1 and CTLA-4 blockade might lead to serious immune-related adverse events (irAEs) such as colitis, hypophysitis, pneumonitis, and thyroiditis [153]. Therefore, ipilimumab is commonly administrated at a reduced dose [154], which might weaken the efficacy of combination therapy. A preclinical study found that prophylactic TNF blockade could dissociate the efficacy and toxicity of α-CTLA-4 plus α-PD-1/PD-L1 therapy [155]. Further clinical investigations are needed to improve the safety and strengthen the efficacy of dual PD-1/PD-L1 and CTLA-4 blockade.

### α-PD-1/PD-L1 plus other ICIs

Other dual immune checkpoint blockade strategies, including α-PD-1/PD-L1 combined with α-TIM-3, α-LAG-3, α-PVRIG, α-TIGIT, are still in clinical trials, having not been approved by the FDA or NMPA. The engagement of TIM-3 with its ligand galectin-9 led to Th1 cell death by triggering intracellular calcium flux [156]. Dual blockade of TIM-3 and PD-1/PD-L1 dramatically enhanced antitumor immune response and retarded tumor growth in murine tumor models [157]. The results of clinical trials showed that α-TIM-3 plus α-PD-1/PD-L1 was tolerable without unexpected safety signals, but more efforts are needed for patient selection [158–160].

Besides α-TIM-3, other ICIs such as α-LAG-3, α-PVRIG, α-TIGIT, α- Siglec-10 also synergized with α-PD-1/PD-L1 in enhancing TIL function and suppressing tumor growth [161–164]. In the phase 2/3 study RELATIVITY-047, relatlimab (α-LAG-3) plus nivolumab therapy demonstrated a significant PFS benefit (10.1 vs. 4.6 months, HR: 0.75) in advanced melanoma, relatively to nivolumab monotherapy [165]. Moreover, in the phase 1 study NCT03667716, COM701 (α-PVRIG) plus nivolumab exhibited encouraging antitumor activity even in some patients with prior ICI treatment [166]. In addition, in the phase 2 study NCT03563716, tiragolumab (α-TIGIT) plus atezolizumab showed an improvement in ORR (OR:2.57, 95%CI:1.07–6.14) and PFS (HR:0.57; 95%CI 0.37–0.90) in PD-L1 positive NSCLC, relative to placebo plus atezolizumab [167].

### Co-stimulatory molecule agonist plus α-PD-1/PD-L1

**Fig. 2:**
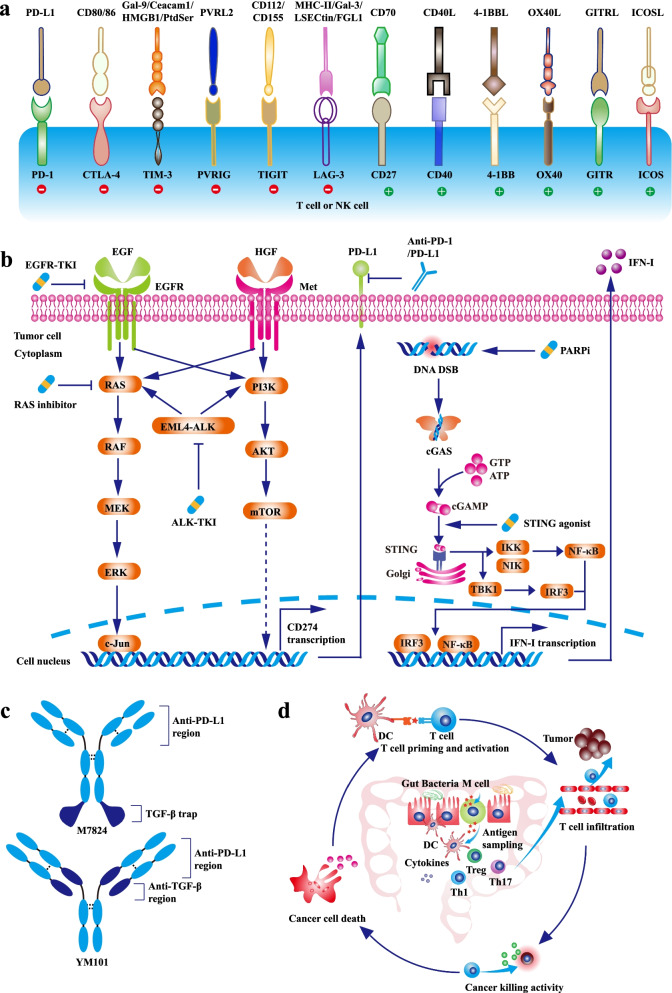
The synergistic antitumor efficacies and mechanisms of α-PD-1/PD-L1 in combination with other novel therapies. **a** The co-inhibitory and co-stimulatory pathways regulating the activities of T cells or NK cells. The green circle refers to co-stimulatory pathway, and the red circle refers to co-inhibitory pathway. **b** Targeted therapy synergizes with α-PD-1/PD-L1. Oncogenic pathways such as MAPK and PI3K-AKT promote PD-L1 transcription. Targeted therapies including EGFR-TKI, ALK-TKI, and RAS inhibitor not only directly retard tumor growth, but also decrease intrinsic PD-L1 expression. Moreover, STING agonist enhances DC function by activating STING-IFN-I pathway. **c** The bifunctional and bispecific antibody containing α-PD-L1 moiety. The structures of M7824 and YM101. **d** The effect of gut microbiota on antitumor immunity. Gut microbiota regulates DC function, Th1-skweing immunity, Th17 polarization, Treg differentiation, and cytokines secretion. Altered gut mucosa immunity could influence the effect of systemic anticancer immunotherapy. Abbreviations: EGFR-TKI, epidermal growth factor receptor-tyrosine kinase inhibitor; ALK, anaplastic lymphoma kinase; PARP, Poly (ADP-ribose) polymerase; DSB, double-strand break; STING, stimulator of interferon genes

Besides co-inhibitory pathways such as PD-1 and CTLA-4, co-stimulatory pathways including CD27/CD70, CD40/CD40L, 4-1BB/4-1BBL, OX40/OX40L, GITR/GITRL, and ICOS/ICOSL also regulate T cell function (Fig. 2a) [168]. Agonists targeting co-stimulatory pathways could enhance T cell activity and revolve antitumor immune response [169]. A series of preclinical studies showed that co-stimulatory molecule agonists improved α-PD-1/PD-L1 efficacy [170–176]. At present, multiple clinical studies of co-stimulatory molecule agonists plus α-PD-1/PD-L1 are ongoing. The preliminary data showed that these combination strategies were well-tolerated, supporting further investigation in advanced solid tumors [177–180].

## Targeted therapy (except for angiogenesis inhibitor) combined with α-PD-1/PD-L1

### Epidermal growth factor receptor-tyrosine kinase inhibitor (EGFR-TKI) plus α-PD-1/PD-L1

EGFR is a member of ErbB family driving the initiation and development of multiple types of cancers [181]. Upon the engagement with its ligands (such as epidermal growth factor, transforming growth factor-alpha, amphiregulin), EGFR would be homodimerized or heterodimerized [182]. Then, the cytoplasmic tyrosine kinases domain of EGFR is phosphorylated, triggering the activation of PI3K-AKT and MAPK pathways [182]. Some cancers especially NSCLC are addicted to the hyperactive EGFR pathway [183]. Therefore, agents targeting EGFR could effectively suppress the growth of these EGFR-addictive cancers. Generally believed, the efficacy of α-PD-1/PD-L1 is modest in *EGFR*-mutated patients [184, 185], which might be attributed to the lack of concurrent TIL and PD-L1 expression, low tumor mutation burden, or increased Tregs in the TME [186]. Recent studies demonstrated that EGFR-TKI could promote T cell infiltration, decrease the ratios of tumor-infiltrating Treg and M2-like macrophage, and improve the responsiveness to α-PD-1/PD-L1 in *EGFR*-mutated models [17, 187]. Besides, activated EGFR signaling contributes to the upregulated PD-L1 on cancer cells, and EGFR-TKI might cooperate with α-PD-1/PD-L1 to attenuate immune evasion [188]. Collectively, EGFR-TKI plus α-PD-1/PD-L1 therapy would maximize the efficacy of immunotherapy in patients with *EGFR*-mutated cancers (Fig. 2b).

In the phase 1 trial CheckMate-012, nivolumab combined with erlotinib showed potent and durable antitumor activity in *EGFR*-mutated NSCLC patients, with tolerable adverse events (no grade 4/5 adverse event reported) (Table 6) [189]. Moreover, in the phase 1 study NCT02013219, *EGFR*-mutated NSCLC patients received erlotinib (150 mg QD for 7 days), followed by erlotinib (150 mg QD) plus 1200 mg atezolizumab (1200 mg, q3w) [190]. The ORR of combination therapy was as high as 75% in the expansion-stage group, and tumor-infiltrating CD8+ T cell was increased in 8/13 paired biopsies after 7-day erlotinib treatment [190]. No pneumonitis and dose-limiting toxicity were reported in this study [190]. However, a retrospective study found that patients receiving nivolumab plus erlotinib might have a higher risk of treatment-associated interstitial pneumonitis (Odds ratio: 4.31, *P* < 0.001), relative to patients undergoing EGFR-TKI monotherapy [191]. Additionally, in the phase 1 study TATTON, the incidence rate of interstitial lung disease in the osimertinib (a third-generation EGFR-TKI) plus durvalumab arm was unexpectedly high (22%), leading to the termination of patient enrollment [192]. Because of the increased risk of treatment-associated interstitial lung disease, a phase 3 clinical trial CAURAL was stopped early [193]. Although the mechanisms of combination therapy-caused irAEs are still unclear, it has been confirmed that treatment sequence and timing are closely associated with the incidence of irAE. PD-1/PD-L1 blockade followed by osimertinib led to a higher incidence rate of irAE, while osimertinib followed by PD-1/PD-L1 blockade decreased the risk of irAE [194]. This phenomenon appears to be unique to osimertinib [194]. The efficacy and toxicity of EGFR-TKI plus α-PD-1/PD-L1 should be further valuated in patients harboring *EGFR*-mutations.

**Table 6:** The clinical trials exploring the efficacy of α-PD-1/PD-L1 combined with targeted therapy (except for angiogenesis inhibitor)

Clinical trial	Phase	α-PD-1/PD-L1	Targeted therapy (Target)	Cancer type	Primary outcome measures
NCT01454102	1	Nivolumab	Erlotinib (EGFR)	NSCLC	Safety
NCT02013219	1	Atezolizumab	Erlotinib (EGFR)	NSCLC	Safety, RP2D
NCT02143466	1	Durvalumab	Osimertinib (EGFR)	NSCLC	Safety
NCT02454933	3	Durvalumab	Osimertinib (EGFR)	NSCLC	Safety
NCT02574078	1/2	Nivolumab	Crizotinib (Met/ALK/ROS)	NSCLC	Safety
NCT02511184	1	Pembrolizumab	Crizotinib (Met/ALK/ROS)	NSCLC	Safety
NCT02013219	1	Atezolizumab	Alectinib (ALK/ FLT3/RET)	NSCLC	Safety, RP2D
NCT02584634	2	Avelumab	Crizotinib (Met/ALK/ROS)	NSCLC	ORR, Safety
NCT02660034	1	Tislelizumab	Pamiparib (PARP)	Solid tumor	Safety, ORR, PFS, DOR, DCR, CBR, OS
NCT04475939	3	Pembrolizumab	Niraparib (PARP)	NSCLC	PFS, OS
NCT02657889	1/2	Pembrolizumab	Niraparib (PARP)	TNBC, Ovarian cancer	Safety, ORR
NCT02734004	2	Durvalumab	Olaparib (PARP)	Ovarian cancer	DCR, ORR, Safety
NCT02484404	1/2	Durvalumab	Olaparib (PARP)	Solid tumor	RP2D, ORR
NCT01988896	1	Atezolizumab	Cobimetinib (MEK)	Solid tumor	Safety, RP2D
NCT02322814	2	Atezolizumab	Cobimetinib (MEK)	TNBC	PFS, ORR
NCT02788279	3	Atezolizumab	Cobimetinib (MEK)	Colorectal cancer	OS
NCT03600883	1/2	Unspecified	AMG 510 (KRAS)	KRAS p.G12C mutant solid tumor	Safety
NCT02972034	1	Pembrolizumab	MK-8353 (ERK)	Solid tumor	Safety
NCT02967692	3	PDR001	Dabrafenib (RAF) and Trametinib (MEK)	Melanoma	Safety, PFS
NCT04017650	1/2	Nivolumab	Encorafenib (RAF) and Cetuximab (EGFR)	Colorectal cancer	Best radiographic response; Safety
NCT03502733	1	Nivolumab	Copanlisib (PI3K)	Solid tumor and lymphoma	Safety
NCT03395899	2	Atezolizumab	Ipatasertib (AKT)	Breast cancer	2-fold increase in GzmB^+^CD8^+^ T cell
NCT02393248	1/2	Pembrolizumab	Pemigatinib (FGFR)	Solid tumor	Maximum tolerated dose, Pharmacodynamics
NCT03123055	1/2	Pembrolizumab	B-701 (FGFR)	Urothelial cell carcinoma	Safety, ORR
NCT02819596	2	Durvalumab	Savolitinib (c-MET)	Renal cell carcinoma	Safety, ORR
NCT02779751	1	Pembrolizumab	Abemaciclib (CDK4/6)	NSCLC, Breast cancer	Safety
NCT04000529	1	Spartalizumab	TNO155 (SHP-2)	Solid tumor	Safety

Abbreviations: *NSCLC* non-small cell lung cancer, *PFS* progression-free survival, *OS* overall survival, *RP2D* recommended phase 2 dose, *DOR* duration of response, *DCR* disease control rate, *CBR* clinical benefit rate

### Anaplastic lymphoma kinase (ALK)-TKI plus α-PD-1/PD-L1

ALK is a receptor tyrosine kinase belonging to insulin receptor superfamily [195]. *EML4-ALK* fusion is the most common ALK arrangement variant in NSCLC patients [196]. The constitutively activated *ALK* fusion gene promotes cancer development by initiating some oncogenic pathways including MAPK, PI3K-Akt, JAK-STAT, and PLCγ [197]. ALK-TKI has dramatically prolonged the survival of *ALK*-arranged patients [198]. Similar to *EGFR*-mutation, *ALK* rearrangement is also related to the poor response to α-PD-1/PD-L1 [199]. A retrospective analysis showed that the co-expression of PD-L1 and CD8 was rare in *ALK*-arranged tumors, which might contribute to the lower response rate to α-PD-1/PD-L1 [200]. Overexpressed ALK fusion protein increased PD-L1 level, promoting the apoptosis of tumor-infiltrating T cells [201]. Besides, ALK inhibition induced immunogenic cell death in *ALK*-arranged cancer cells and conferred the protection of tumor rechallenge in the mouse model [202]. Combination therapy of α-PD-1 and ceritinib had an enhanced antitumor efficacy in *NPM1-ALK*^+^ R80 model [202].

It should be noted that ALK-TKI combined with α-PD-1/PD-L1 might increase treatment-associated hepatotoxicity. In the phase 1/2 study CheckMate-370, 38% of patients receiving nivolumab plus crizotinib developed severe hepatic toxicities, leading to the termination of the enrollment [203]. Moreover, pembrolizumab plus crizotinib also showed intolerable hepatotoxicity in NSCLC [204]. Conversely, some other combination strategies such as atezolizumab plus alectinib and avelumab plus lorlatinib had a manageable safety profile, indicating the hepatotoxicity might be ALK-TKI specific [205, 206]. Additionally, the timing and sequence of combination therapy also influence treatment toxicity, which should be further validated in clinical studies [186, 207].

### RAS-targeted therapy plus α-PD-1/PD-L1

*RAS* family (*KRAS*, *NRAS* and *HRAS*) is frequently mutated in cancer cells. Mutated *KRAS* is a well-established driver gene of NSCLC, colorectal cancer, and pancreatic cancer [208]. In normal cells, RAS is activated by growth factor receptors such as EGFR. RAS is a small G protein, toggling between GTP-bound state (active) and GDP-bound state (inactive). In active state, RAS triggers several downstream pathways including MAPK and PI3K-AKT [209]. In tumor cells, mutations in RAS disturb this switch between GTP-bound state and GDP-bound state. As a result, RAS is locked in GTP-bound state, leading to the hyperactive downstream pathways and tumor growth [209, 210]. Recent studies have shown that RAS and its downstream pathways participated in cancer immune escape: negatively regulating MHC-I expression on cancer cells, increasing the cell-intrinsic PD-L1 level, elevating immune suppression-associated cytokine production [211, 212]. RAS-targeted therapy abrogated RAS-MAPK/PI3K-AKT-involved immune evasion, synergizing with α-PD-1/PD-L1 [213, 214].

In the phase 1 study NCT01988896, atezolizumab plus cobimetinib (MEK inhibitor) had a manageable safety profile and clinical activity in advanced solid tumors, regardless of KRAS/BRAF status [215]. However, in the phase 2 study NCT02322814, atezolizumab plus cobimetinib and taxane had no improvement in ORR in TNBC, relative to cobimetinib plus taxane [216]. Moreover, in the phase 3 study NCT02788279 exploring the efficacy of atezolizumab plus cobimetinib in metastatic colorectal cancer, the primary endpoint of improved OS (atezolizumab plus cobimetinib vs. regorafenib) could not be reached [217]. At present, other combination strategies including α-PD-1/PD-L1 plus AMG 510 (RAS inhibitor) (NCT03600883), MK-8353 (ERK inhibitor) plus pembrolizumab (NCT02972034), PDR001 (α-PD-1) plus dabrafenib (RAF inhibitor) and trametinib (MEK inhibitor) (NCT02967692), nivolumab plus encorafenib (RAF inhibitor) and cetuximab (α-EGFR) (NCT04017650), nivolumab plus copanlisib (PI3K inhibitor) with or without ipilimumab (NCT03502733), atezolizumab plus ipatasertib (AKT inhibitor) (NCT03395899) are still in clinical trials [218].

### Poly (ADP-ribose) polymerase (PARP) inhibitor plus α-PD-1/PD-L1

Normal cells preferentially repair double strand break (DSB) via homologous recombination (HR). However, some HR-deficient (e.g. *BRCA1/2* mutant) cancer cells only repair DSB by nonhomologous end joining, which is a low fidelity repair pathway [219]. As a result, chromosomal rearrangements are accumulated in cancer cells, eventually leading to cell death [220]. Therefore, intact single-strand break (SSB) repair pathway is essential to these HR-deficient cancer cells. Based on this synthetic lethality theory, interfering SSB could destroy HR-deficient cancer cells [221]. As the core of SSB repair, PARP is the ideal target for drug development [222]. Besides synthetic lethal effect, PARP inhibitor (PARPi) modulates the TME and promotes the antitumor immune response [223]. Firstly, PARPi activates cGAS-STING pathway in cancer cells and increases T cell recruitment [224]. Moreover, PARPi upregulates PD-L1 expression by inactivating GSK3β signaling, which attenuates antitumor immunity [225]. Inspired by the results of preclinical studies, numerous clinical studies are ongoing to evaluate the efficacy of PARPi combined with α-PD-1/PD-L1 [219].

In the phase 1 study NCT02660034, pamiparib plus tislelizumab was well-tolerated, and 20% of patients with advanced solid tumors achieved an objective response at a median follow-up of 8.3 months [226]. Additionally, in the phase 2 study JASPER, niraparib plus pembrolizumab exhibited a powerful antitumor activity especially in PD-L1^high^ (tumor proportion scores TPS ≥ 50%) advanced NSCLC patients (ORR: 56.3%) [227]. Moreover, in the phase 1/2 study KEYNOTE-162, niraparib plus pembrolizumab was tolerable, with a considerable antitumor efficacy in recurrent ovarian carcinoma (ORR: 18%; DCR: 65%) [228]. Besides, olaparib and durvalumab arm also had a higher ORR than that reported for PARPi treatment in germline BRCA-mutated platinum-sensitive relapsed ovarian cancer [229]. Accumulating evidence indicates that PARPi plus α-PD-1/PD-L1 is a promising combination strategy in multiple types of cancers, including metastatic castrate-resistant prostate cancer and metastatic TNBC [230, 231].

### α-PD-1/PD-L1 plus other novel targeted therapies

Dysregulated fibroblast growth factor-fibroblast growth factor receptor (FGF-FGFR) signaling participates in cancer development by activating MAPK, PI3K, and PLC-γ pathways [232, 233]. Mutant FGFR signaling might be related to the poor response to α-PD-1/PD-L1, and FGFR inhibitor synergized with α-PD-1/PD-L1 in FGFR^mut^ models [234]. The combination of erdafitinib (FGFR inhibitor) and α-PD-1 broadened the TCR repertoire and increased T cell fraction, contributing to the superior antitumor efficacy [234]. Besides, lenvatinib (VEGFR/FGFR inhibitor) plus α-PD-1 also showed a synergistic antitumor effect in the murine HCC model [235]. The clinical studies exploring the efficacy of FGFR inhibitor plus α-PD-1 are still undergoing. The interim results of phase 1/2 study NCT02393248 indicated pemigatinib (FGFR inhibitor) combined with pembrolizumab therapy was tolerable, with a potent antitumor effect in FGFR^mut^ patients [236]. Besides, the preliminary results of phase 1/2 study NCT03123055 demonstrated that vofatamab (FGFR inhibitor) plus pembrolizumab had an encouraging effect in FGFR^WT^ metastatic urothelial carcinoma [237].

c-MET is also known as hepatocyte growth factor receptor (HGFR). Activated c-MET signaling triggers downstream MAPK, PI3K-AKT, RAC1, and FAK pathways [238]. c-MET signaling is hyperactivated in multiple cancers, due to *MET* mutations, amplification, or rearrangement [238]. c-MET signaling upregulated PD-L1 expression, and c-MET inhibitor impaired intrinsic and IFN-γ-induced PD-L1 expression [239–241]. In the phase 2 study NCT02819596, savolitinib (c-MET inhibitor) plus durvalumab had clinical activity in MET-driven papillary renal cancer (Confirmed RR: 57%, median PFS: 10.5 months, median OS: 27.4 months) [242].

Cyclin-dependent kinase 4/6 (CDK4/6) is an essential component of cell cycle, which cooperates with cyclin D to promote cell cycle G1/S transition [243]. CDK4/6 inhibitors suppress tumor growth by cell cycle arrest [243]. Besides interfering cell division, CDK4/6 inhibitors also had immunomodulatory activity. CDK4/6 inhibitors promoted NF-κB activation, increased T cell chemoattractant and PD-L1 level, and prevented PD-L1 degradation in cancer cells [244]. Besides, CDK4/6 inhibitors upregulated NFAT activity and elevated effector gene expression in T cells [244]. CDK4/6 inhibitors improved the efficacy of α-PD-1 in murine models by enhancing lymphocyte infiltration and TIL activities [245–248]. The interim data of phase 1b study NCT02779751 showed that abemaciclib (CDK4/6 inhibitor) plus pembrolizumab had antitumor activity in *KRAS*^mut^ non-squamous NSCLC [249].

SHP2 is an oncogenic protein belonging to protein tyrosine phosphatases family [250]. As the convergent node of MAPK, PI3K-AKT, JAK-STAT, and PD-1 pathways, SHP2 widely regulates multiple cancer-associated processes such as cell survival and immune escape [251]. SHP2 inhibition increased PD-L1 and MHC-I expression by augmenting intrinsic IFN-γ in cancer cells [252]. SHP2 inhibitor enhanced the efficacy of α-PD-1 in murine tumor models [252–254]. A clinical study exploring SHP2 inhibitor combined with α-PD-1 is still ongoing (NCT04000529), and the final data of this combination study are not yet available [255].

## STING agonist plus α-PD-1/PD-L1

### STING pathway and STING agonist

Cytosolic chromatin fragments and micronuclei are commonly accumulated during malignant transformation, increasing the probability of cytosolic DNA leakage in cancer cells or tumor-derived DNA uptake in DCs [256]. cGAS-STING pathway is a cytosolic DNA sensing signaling. Cytosolic dsDNA binds to cGAS, catalyzing the generation of cyclic GMP-AMP (cGAMP). Stimulated by cGAMP, STING changes from monomer to dimer and translocates from ER to perinuclear microsome. Then, STING recruits and phosphorylates TBK1, which further activates downstream IRF3 and upregulates IFN-I [257–259]. Besides, STING also increases IFN-I by activating NF-κB pathway [260]. IFN-I is a versatile immune stimulator that could enhance the functions of DC, NK, and T cells [261]. Given the critical role of cGAS-STING pathway in bridging innate and adaptive immunity, STING is the potential target for cancer immunotherapy.

Dimethyloxoxanthenyl acetic acid (DMXAA) is the first STING agonist which failed in the clinical trials [262]. Further investigation has identified that DMXAA is a mouse-specific STING agonist, with a subtle influence on human STING pathway [263, 264]. Sharing similar structures and biological characteristics with cGAMP, some natural and artificially synthetic cyclic dinucleotides (CDNs) are developed as STING agonists for cancer immunotherapy [265–267]. Generally, CDNs have two main flaws: poor transmembrane capability and depending on intratumor injection. Recently, some novel STING agonists such as diABZI and MSA-2 have been developed which could be systemically administrated [268, 269]. Besides, manganese is also identified as a natural STING agonist, playing an important role in antitumor immunity [270, 271].

### STING agonist plus α-PD-1/PD-L1

The combination therapy of STING agonist and α-PD-1/PD-L1 simultaneously boosts innate immunity and adaptive immunity, effectively overcoming resistance to immunotherapy. On the one hand, STING agonist promotes immune cell infiltration and enhances the function of APC, NK, and T cells [272–274]. On the other hand, α-PD-1/PD-L1 antibodies take advantage of STING agonist-induced PD-L1 upregulation [273]. Up to now, multiple clinical trials of STING agonist combined with α-PD-1/PD-L1 are ongoing. The preliminary data showed that some combination therapies (e.g. ADU-S100 plus spartalizumab, MK-1454 plus pembrolizumab, manganese plus α-PD-1) had encouraging antitumor activity with a tolerable safety profile [270, 275, 276].

## Bispecific/bifunctional antibody targeting PD-1/PD-L1

Dual targeting by bispecific/bifunctional antibodies has emerged as an option for combination therapy. Bispecific/bifunctional antibody simultaneously blocks two molecules with one drug, having a strategic advantage over the combination therapy (Table 7) [277].

**Table 7:** Bispecific/bifunctional antibodies targeting PD-1/PD-L1

Target	Antibody Product	Company/Authors	Reference
TGF-β × PD-L1	M7824	Merck KGaA	[278]
	YM101	Wuhan YZY Biopharma	[279]
	SHR-1701	Hengrui Pharmaceuticals	[280]
CTLA-4 × PD-L1	KN046	Alphamab Oncology	[281]
CTLA-4 × PD-1	MGD019	MacroGenics	[282]
	MEDI5752	AstraZeneca	[283]
LAG-3 × PD-L1	IBI323	Innovent Biologics	[284]
LAG-3 × PD-1	Tebotelimab	MacroGenics	[285]
TIM-3 × PD-L1	LY3415244	Eli Lilly	[286]
TIGIT×PD-L1	Not given	Novamab Biopharmaceuticals	[287]
4-1BB × PD-L1	MCLA-145	Merus and Incyte	[288]
	ABL503	ABL Bio	[289]
	PM1003	Biotheus	[290]
CD27 × PD-L1	CDX-527	Celldex Therapeutics	[291]
c-Met×PD-1	Not given	Yuan et. al	[292]
	Not given	Hou et. al	[293]
	Not given	Wu et. al	[294]
	Not given	Sun et. al	[295]
EGFR×PD-L1	Not given	Koopmans et. al	[296]
PD-1 × PD-L1	LY3434172	Eli Lilly	[297]
CD47 × PD-L1	IBI322	Innovent Biologics	[298]

### TGF-β × PD-L1 bispecific/bifunctional antibody

TGF-β is a well-studied immunoinhibitory cytokine: restraining immune cell infiltration, inducing Treg differentiation, and hampering the functions of T cell, APC, and NK [17]. Hyperactivated TGF-β signaling was associated with the poor response to α-PD-1/PD-L1, and blocking TGF-β significantly improved the efficacy of α-PD-1/PD-L1 [299–302]. M7824 is a TGF-β × PD-L1 bifunctional protein, which had potent antitumor activity in murine tumor models (Fig. 2c) [278]. The results of phase 1 studies were encouraging, and patients receiving M7824 had a higher ORR, compared to previous data [303, 304]. Besides, in the phase 1 study NCT03710265, SHR-1701 (TGF-β × PD-L1 bifunctional antibody) showed encouraging antitumor activity [280]. Apart from bifunctional antibody, the TGF-β × PD-L1 bispecific antibody YM101 also exhibited robust antitumor activity in immune-excluded tumor models [279]. Further investigation showed that YM101 promoted T cell infiltration, enhanced T cell function, impaired cancer-associated fibroblasts (CAF) activity, and induced macrophage polarization toward M2-like phenotype [279]. The antitumor mechanisms of TGF-β × PD-L1 bispecific/bifunctional antibody are well-understood, thus an enormous number of resources are spent on the development of analogical antibodies.

### Bispecific antibody targeting two inhibitory immune checkpoints

The resistance to α-PD-1/PD-L1 is related to the upregulation of other immune checkpoints. Therefore, bispecific antibodies targeting two inhibitory immune checkpoints might relieve α-PD-1/PD-L1 resistance. Numerous bispecific antibodies have been developed, including CTLA-4 × PD-L1 (KN046) [281], CTLA-4 × PD-1 (MGD019 and MEDI5752) [282, 283], LAG-3 × PD-L1 (IBI323) [284], LAG-3 × PD-1 (Tebotelimab) [285], TIM-3 × PD-L1 (LY3415244) [286], TIM-3 × PD-1 [305], TIGIT×PD-L1 [287]. Most bispecific antibodies achieved excellent antitumor efficacies in murine tumor models. Some bispecific antibodies have been in clinical trials, showing preliminary antitumor activity.

### Bispecific antibody targeting PD-1/PD-L1 and co-stimulatory molecules

As mentioned above, agonists targeting co-stimulatory molecules synergize α-PD-1/PD-L1. It is rational to develop bispecific antibodies targeting PD-1/PD-L1 and co-stimulatory molecules to optimally engage antitumor immune response. Multiple bispecific antibodies have been successfully constructed, including 4-1BB × PD-L1 (MCLA-145, ABL503, PM1003) [288–290] and CD27 × PD-L1 (CDX-527) [291]. These bispecific antibodies augmented the functions of TILs and exerted a powerful antitumor efficacy [288–291].

### Other bispecific antibodies targeting PD-1/PD-L1

The synergistic effect between c-MET inhibitor and α-PD-1/PD-L1 has been verified [306]. c-MET×PD-1 bispecific antibodies simultaneously reversed c-Met-mediated cell proliferation and migration and enhanced T cell functions [292–295]. Moreover, avoiding ‘on-target/off-tumor’ binding to PD-L1 on nonmalignant cells, EGFR×PD-L1 bispecific antibody was developed for EGFR+ tumors [296]. This antibody had an enhanced tumor specificity, reducing the risks of the indiscriminate reactivation of antitumor T cells and severe treatment-related adverse events [296]. These tumor-associated antigen×PD-1/PD-L1 bispecific antibodies might have a great advantage in efficacy and safety. Besides, PD-1 × PD-L1 (LY3434172) and CD47 × PD-L1 (IBI322) bispecific antibodies had enhanced immunomodulatory properties and improved antitumor activity, relative to monospecific PD-1 and PD-L1 antibodies [297, 298].

## Other novel combination strategies

### FMT plus α-PD-1/PD-L1

The influence of gut microbiota on host immunity is multifaceted, simultaneously regulating the gut mucosal immune system and systemic immune system (Fig. 2d) [307–310]. It was reported that the gut microbiota of immunotherapy-sensitive patients was distinct from that of immunotherapy-resistant populations [311, 312]. Some specific bacteria including *Bifidobacterium*, *Faecalibacterium*, *Akkermansia muciniphila*, and *Bacteroides fragilis* enhanced the functions of DC and T cells, contributing to the better response to immunotherapy [313]. Conversely, bacteria including *Bacteroidales*, *Ruminococcus obeum*, and *Roseburia intestinalis* increased the immunoinhibitory components such as MDSC and Treg, impairing the efficacy of immunotherapy [313]. Besides, manipulating gut microbiota composition could improve the response to α-PD-1/PD-L1 [311, 314–316]. The results of two phase 1 clinical studies NCT03341143 and NCT03353402 showed responder-derived FMT effectively relieved the resistance to α-PD-1/PD-L1 in some melanoma patients, having implications for modulating gut microbiota in cancer immunotherapy [26, 317].

### Immunostimulatory cytokine treatment or immunoinhibitory cytokine blockade plus α-PD-1/PD-L1

Some cytokines including GM-CSF, IFN-α, IL-2, IL-7, IL-12, IL-15, IL-18, and IL-21 have antitumor activity via stimulating immunity, inhibiting proliferation, or inducing apoptosis in cancer cells [318]. Moreover, neutralizing cytokines including TGF-β and IL-6 potentiates antitumor immunity [319, 320]. The safety and efficacy of α-PD-1/PD-L1 combined with IL-2 pathway agonist (NKTR-214/BEMPEG) [321], PEGylated IL-10 (Pegilodecakin) [322], IL-12 plasmid (Tavo) [323], IL-15 agonist (ALT-803) [324], or PEGylated IFN-α [325] had been validated in cancer patients. The preliminary results support further clinical trials to assess the optimal sequencing and combination of α-PD-1/PD-L1 and cytokine therapy.

### Epigenetic modifiers plus α-PD-1/PD-L1

Epigenetic alterations such as histone acetylation regulate PD-L1 expression [188]. Beyond direct cytotoxicity, histone deacetylases (HDAC) inhibitors changed immunogenicity and enhanced antitumor immunity, via decreasing MDSC ratio and upregulating MHC-I/II, CD40, CD80, and CD86 expression [326–328]. HDAC inhibitor combined with α-PD-1/PD-L1 has shown a synergistic antitumor effect in murine tumor models [329–331]. Inspired by the encouraging results of preclinical studies, the combination therapy of entinostat (HDAC1/3 inhibitor) and pembrolizumab is in clinical trials. The preliminary results (NCT02697630) showed that entinostat plus pembrolizumab induced durable responses in some patients with metastatic uveal melanoma, and more entinostat-involved combination regimes such as entinostat plus avelumab (NCT02915523), nivolumab (NCT03838042), or M7824 (NCT04708470) are still in clinical evaluation [332].

### Metabolic modulators plus α-PD-1/PD-L1

The engagement of adenosine 2A receptor (A2AR) with adenosine elicits immunoinhibitory effects: suppressing the activities of tumor-infiltrating CD8+ T cells and hampering the function and differentiation of DCs [333, 334]. The accumulated adenosine in the TME promotes cancer immune evasion, and A2AR blockade rescues immune cell function [335, 336]. The results of a phase 1 study showed that ciforadenant (A2AR inhibitor) combined with atezolizumab effectively prolonged PFS and OS in RCC patients [337]. Besides, other metabolic modulators such as glutaminase inhibitor also had a synergistic effect with α-PD-1/PD-L1 in murine tumor models [338].

### Chimeric antigen receptor-T (CAR-T) cell therapy plus α-PD-1/PD-L1

CAR-T cells are genetically engineered T cells, which could recognize and bind cancer antigen in an MHC-independent manner [339]. CAR-T cell therapy provides numerous cancer-reactive T cells and overcomes MHC downregulation-mediated cancer immune evasion [339]. However, the efficacy of CAR-T cell therapy is modest in most solid tumors, which is partly attributed to the immunosuppressive TME [340]. α-PD-1/PD-L1 enhanced CAR-T cell therapy by rescuing CAR-T cell exhaustion [341–343]. The results of phase 1 study demonstrated CAR-T cell therapy combined with α-PD-1/PD-L1 had confirmed antitumor activity in patients with malignant pleural diseases [344]. Moreover, modified CAR-T cells, which secret PD-1-blocking single-chain variable fragments (scFv), had improved antitumor activity by an autocrine and paracrine manner [345]. This combination strategy protects CAR-T cells from immune exhaustion and optimizes CAR-T cell efficacy.

## Perspective and conclusion

Although dozens of combination regimens exhibit potent antitumor activities in preclinical studies, some positive preclinical findings could not be validated in the clinic. At present, only combinations of α-PD-1/PD-L1 with chemotherapy, angiogenesis inhibitor, or α-CTLA-4 are approved by the FDA or NMPA. For most combinations, the striking antitumor activities are limited in animal tumor models. Therefore, how to select an optimal preclinical model is a grand challenge to identify the activities of combination regimens. Relative to widely used syngeneic murine models, humanized patient-derived models could provide a more precious efficacy evaluation. Besides, combination therapy increases the risk of irAEs and the cost of health care. Inappropriate combination treatments will expose patients to significantly higher toxicities. How to optimize administration regimen, including dosage, timing, and sequence, is another challenge for the development of combination therapy. Lastly, it is still unclear how to select appropriate combination therapy and find biomarkers predicting treatment response. Considering the heterogeneity and evolution of tumors, liquid biopsy could dynamically monitor the immune landscape of the TME and provide a real-time biomarker for guiding precision immunotherapy [346]. We believe individualized combination therapy should be provided based on patient’s immune profiling and other predictive biomarkers. A comprehensive framework integrating genome, transcriptome, immune profiling, microbiome could be adopted to select patients benefiting from combinations.

**Fig. 3:**
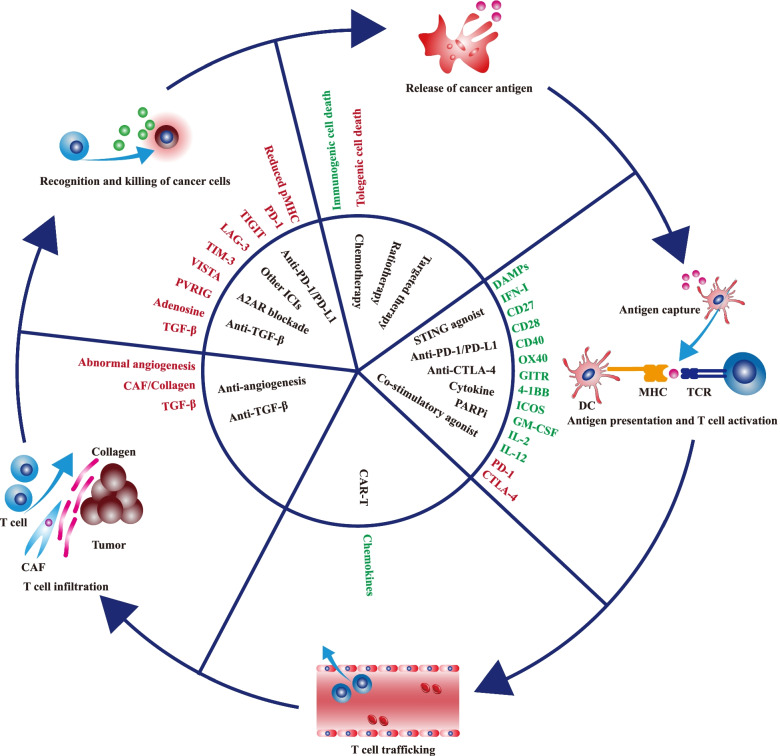
Therapies regulating the cancer-immunity cycle. The cancer-immunity cycle starts with cancer antigen release and ends with cancer cell-killing by immune cells. Each step in the cycle is regulated by various factors. The stimulatory factors (shown in green) enhance antitumor immunity, while the inhibitory factors (shown in red) undermine antitumor immunity. These factors provide potential therapeutic targets to promote antitumor immunity. The figure presents some of therapies regulating the cancer-immunity cycle. Abbreviations: CAF, cancer-associated fibroblasts; PARP, Poly (ADP-ribose) polymerase; DSB, double-strand break; STING, stimulator of interferon genes; A2AR, adenosine 2A receptor

For patients with non-inflamed tumors, α-PD-1/PD-L1 monotherapy scarcely provides clinical benefits, and a personalized combination is needed to overcome drug resistance. In the background of immune-excluded, therapies such as TGF-β blocker could rescue the restrained T cell penetration by inhibiting CAF activities and reducing peritumoral collagen deposition. In the context of immune-desert, therapies such as radiotherapy, chemotherapy, and STING agonist could overcome low immunogenicity-mediated immune tolerance by inducing immunogenic cell death, increasing cancer antigen release, and promoting the function of APC. Combining these therapies with α-PD-1/PD-L1 simultaneously boosts multiple processes in the cancer-immunity cycle, reshapes the TME, and substantially promotes the transformation from non-inflamed to inflamed tumors (Fig. 3). Besides, with the development of next-generation α-PD-1/PD-L1 drugs such as bifunctional or bispecific antibodies, the indication of α-PD-1/PD-L1 therapies would be greatly extended, and more patients could benefit from the updated α-PD-1/PD-L1 treatments.

## Data Availability

Not applicable.

## Abbreviations

PD-1: Programmed cell death 1
TCR: T cell receptor
SHP-2: Src homology region 2 domain-containing phosphatase
pAPC: Professional antigen presentation cell
STING: Stimulator of interferon genes
FMT: Fecal microbiota transplant
TME: Tumor microenvironment
DAMP: Damage-associated molecular pattern
CRT: Calreticulin
HMGB1: High-mobility group box 1
DC: Dendritic cell
Treg: Regulatory T cell
TAM: Tumor-associated macrophage
MDSC: Myeloid-derived suppressor cell
TIL: Tumor-infiltrating lymphocyte
PFS: Progression-free survival
OS: Overall survival
ORR: Objective response rate
NSCLC: Non-small cell lung cancer
GEJC: Gastroesophageal junction cancer
TNBC: Triple-negative breast cancer
FDA: Food and drug administration
NMPA: National medical products administration
SBRT: Stereotactic body radiotherapy
VEGF: Endothelial growth factor
ANGPT2: Angiopoietin 2
PLGF: Placental growth factor
EGFR-TKI: Epidermal growth factor receptor-tyrosine kinase inhibitor
irAE: Immune-related adverse event
ALK: Anaplastic lymphoma kinase
PARP: Poly (ADP-ribose) polymerase
DSB: Double strand break
HR: Homologous recombination
SSB: Single strand break
FGFR: Fibroblast growth factor receptor
HCC: Hepatocellular carcinoma
HGFR: Hepatocyte growth factor receptor
CDK4/6: Cyclin-dependent kinase 4/6
cGAMP: Cyclic GMP-AMP
DMXAA: Dimethyloxoxanthenyl acetic acid
CDN: Cyclic dinucleotide
CAF: Cancer-associated fibroblast
HDAC: Histone deacetylase
A2AR: Adenosine 2A receptor
CAR-T: Chimeric antigen receptor T
